# A Comparison between Revised NCEP ATP III and IDF Definitions in Diagnosing Metabolic Syndrome in an Urban Sri Lankan Population: The Ragama Health Study

**DOI:** 10.1155/2013/320176

**Published:** 2013-02-27

**Authors:** S. Chackrewarthy, D. Gunasekera, A. Pathmeswaren, C. N. Wijekoon, U. K. Ranawaka, N. Kato, F. Takeuchi, A. R. Wickremasinghe

**Affiliations:** ^1^Faculty of Medicine, University of Kelaniya, 11010 Ragama, Sri Lanka; ^2^Department of Gene Diagnostics and Therapeutics, Research Institute, National Center for Global Health and Medicine, 1-21-1 Toyama, Shinjuku-ku, Tokoyo 162-8655, Japan

## Abstract

*Background*. The prevalence of metabolic syndrome (MetS) within individual cohorts varies with the definition used. The aim of this study was to compare the prevalence of MetS between IDF and revised NCEP ATP III criteria in an urban Sri Lankan population and to investigate the characteristics of discrepant cases. *Methods*. 2985 individuals, aged 35–65 years, were recruited to the study. Anthropometric and blood pressure measurements and laboratory investigations were carried out following standard protocols. *Results*. Age and sex-adjusted prevalences of MetS were 46.1% and 38.9% by revised NCEP and IDF definitions, respectively. IDF criteria failed to identify 21% of men and 7% of women identified by the revised NCEP criteria. The discrepant group had more adverse metabolic profiles despite having a lower waist circumference than those diagnosed by both criteria. *Conclusion*. MetS is common in this urban Sri Lankan cohort regardless of the definition used. The revised NCEP definition was more appropriate in identifying the metabolically abnormal but nonobese individuals, especially among the males predisposed to type 2 diabetes or cardiovascular disease. Further research is needed to determine the suitability of the currently accepted Asian-specific cut-offs for waist circumference in Sri Lankan adults.

## 1. Introduction

Metabolic syndrome (MetS) is a complex web of metabolic risk factors that are associated with a 5-fold risk of type 2 diabetes (T2DM) and a 2-fold risk of cardiovascular disease (CVD) [1, 2]. Individuals with MS have a 30–40% probability of developing diabetes and/or CVD within 20 years, depending on the number of components present [3]. Although there are different definitions of MetS, the uniform pathophysiology of this syndrome is insulin resistance [4]. MetS appears to have a component of heritability, which suggests a genetic basis [5]. However, the association is complex, and the role of gene-environment interactions, ethnicity, and gender in the pathogenesis of MetS needs to be further explored.

The prevalence of MetS increases with altered glucose metabolism [6] and with the increasing worldwide prevalence of T2DM, the expected increase in the frequency of occurrence of MS will rise to alarming proportions. South Asians have an unusually high tendency to develop T2DM and coronary heart disease (CHD) [7–9]. Higher prevalence, earlier onset, and increased complications of T2DM and CHD are often seen at lower levels of body mass index (BMI) and waist circumference (WC) in South Asians than in white Caucasians [10, 11]. It was estimated that 20%–25% of South Asians have developed MetS, and many more may be prone to it [12].

Prevalence of diabetes in Sri Lanka is one of the highest in the South Asia [12]. A study on a nationally representative sample reported a current diabetes prevalence of 10.3% (9.8% for men and 10.9% for women) [13]. CHD (10.6%) is the leading cause of death in the country (by percentage of the total mortality for 2000) [14]. Given that MetS is a strong predictor of T2DM and CVD, a higher prevalence of MetS can be suspected among Sri Lankans. However, limited data are available on the prevalence of MetS in Sri Lanka, and there is a paucity of studies comparing the different diagnostic criteria with ethnic and gender-specific cut-off values for abdominal obesity as recommended by the International Diabetes Federation (IDF) [15] and revised National Cholesterol Education Program Adult Treatment panel III (revised NCEP ATP III) [16].

The aim of the present study was to determine the prevalence of MetS in an urban Sri Lankan population according to gender, age, and glycaemic status using IDF and revised NCEP-ATP III guidelines using ethnic and gender-specific waist circumference cut-off values and to investigate the concordance between the two definitions to demonstrate if the participants identified by revised NCEP ATP III and not by the IDF had the same metabolic risk profile. 

## 2. Materials and Methods

Ragama Health Study (RHS) is a collaborative effort between the Faculty of Medicine, University of Kelaniya, Sri Lanka and the International Medical Centre of Japan (IMCJ). Ethical approval was obtained from Ethical Review Committees of both institutions. This study was conducted in 2007, in the Ragama MOH area, an urban community situated 18 Km north of the capital city Colombo. 

### 2.1. Study Population

A total of 2985 adults (1358 men and 1627 women) aged between 35 and 65 years constituted the study population. The householders list of each administrative division in the Ragama MOH area was used as the sampling frame, and the study population was stratified into 35–44, 45–54, and 55–65 years. A random sample of 200 adults was obtained from each administrative division, in a ratio of 1 : 2 : 2 in the age groups of 35–44, 45–54, and 55–65, respectively. All selected participants were visited at their homes and invited to participate in the study.

### 2.2. Measurements and Assays

Selected participants were requested to present at a special clinic set up at the Faculty of Medicine, University of Kelaniya. Informed written consent was obtained, and detailed questionnaires were administered for information regarding demographic, socioeconomic, nutritional, and health status of participants.

Anthropometric measurements including weight, height, waist, and hip measurements were obtained using standardized techniques. Blood pressure was measured from the right upper limb in the sitting position using an Omron 705CP automatic blood pressure monitor. The mean value of two readings taken five minutes apart was recorded. 

Fasting plasma glucose was measured according to the hexokinase method, and HbA_1c_ was measured by ion exchange HPLC (Variant, Bio-Rad, USA). Serum insulin levels were assayed by enzyme immunoassay (Abbott Diagnostics, Germany). Serum total cholesterol was measured by the method described by Stadtman [17]. Triglycerides were measured by standard enzymatic methods using TGL Flex reagent cartridges, and HDL-C was measured using AHDL-Flex reagent cartridges on Dade Behring Dimension Clinical Chemistry System, USA. Insulin resistance was evaluated by the homeostasis model assessment of insulin resistance (HOMA-IR) [18].

Impaired fasting glucose (IFG) was defined according to 2004 ADA definition [19] as fasting plasma glucose (FPG) ≥100 mg/dL to <126 mg/dL (5.6 mmol/L–7.0 mmol/L), and individuals with a FPG ≥126 mg/dL or history of diabetes or on glycaemic medication were classified as having diabetes regardless of the measured FPG values. 

### 2.3. Definitions of Metabolic Syndrome

We compared the IDF criteria for MetS with the 2005 revised NCEP ATP III criteria as proposed by the AHA/NHLB. The revised NCEP criteria (16) require at least three of the following components: (1) abdominal obesity (waist circumference ≥90 cm for Asian men or ≥80 cm for Asian women), (2) triglycerides ≥150 mg/dL, (3) HDL cholesterol ≤40 mg/dL for men or 50 mg/dL for women, (4) systolic/diastolic blood pressure ≥130/85 mmHg or receiving drug treatment, and (5) fasting plasma glucose ≥100 mg/dL. For NCEP criteria abdominal obesity is a component of the syndrome but not a prerequisite for diagnosis. The IDF criteria of MetS (15) uses central obesity (waist circumference ≥90 cm for South Asian men or ≥80 cm for South Asian women) as a mandatory criterion and the presence of at least two of the other four criteria which are identical to those provided by NCEP ATP III. 

### 2.4. Statistical Analysis

Prevalence of MetS was calculated by gender, age, and diabetic status. Adjusted prevalences were calculated using sampling weights to ensure that the estimated prevalence was representative of the Ragama MOH area. The differences in the prevalence of metabolic abnormalities between genders were assessed using the *Z* test for difference between proportions. The kappa statistic (*κ*) was used to determine the agreement between the two diagnostic criteria. The analysis was carried using Stata version 8.

## 3. Results

A total of 2985 adults aged between 35 and 65 years participated in the study with 1627 (54.5%) females. 83% of the study population was aged 45 years or above. 

The age-sex-standardized prevalence of MetS according to revised NCEP and IDF definitions were 46.1% (95% CI, 45.8–46.4%) and 38.9% (95% CI, 37.2–40.7%), respectively (Table 1). There were 133 (74 males and 59 females) or 11.5% of all participants who were diagnosed by the revised NCEP definition but missed by the IDF definition. Those participants missed by the IDF definition were mainly males (55.6%). There were no participants who were diagnosed by IDF but missed by the revised NCEP definition. Among those diagnosed to have metabolic syndrome, 88.5% of participants were identified equally by both definitions. The agreement between these two definitions as shown by the Kappa statistic was 0.84 ± 0.01 for the total population, 0.87 ± 0.01 for women, and 0.78 ± 0.02 for men.

Prevalence of MetS was age dependent (Table 1). In women, the 45–55 year age group showed a marked increase in the prevalence of MetS, and more than 50% above the age of 45 years were identified as having MetS by either definition. In men, however, the increase in prevalence between age groups was not significant, indicating that changes in the metabolic profile occurred at a slower pace. With regards to gender, the prevalence of MetS was significantly higher in females than in males by both revised NCEP (56.1% versus 33.9%, *P* < 0.001) and IDF (45.8% versus 22.9%, *P* < 0.001) definitions. 

**Figure 1:**
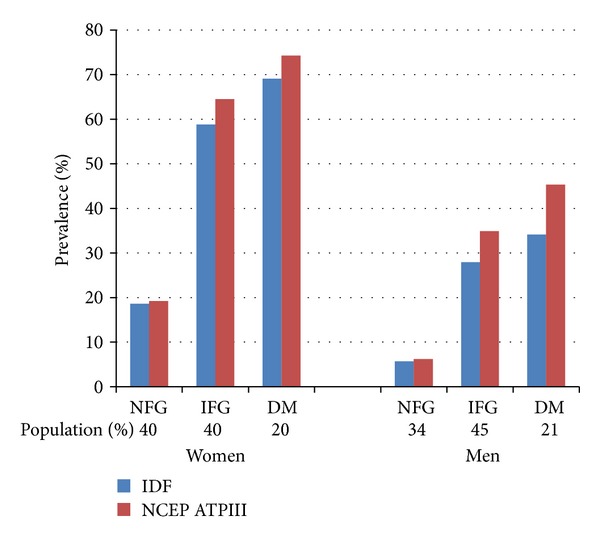
Gender-specific crude prevalences of metabolic syndrome categorized by glycaemic status (NFG: normal fasting glucose, IFG: impaired fasting glucose, and DM: diabetes mellitus).

Age-adjusted prevalence of MetS in the cohort categorized according to glycogenic status is shown in Figure 1. There is a stepwise increase in the prevalence of MetS by either definition with the worsening glycaemic status. This effect was more marked in the transition from normal fasting glucose status (NFG) to impaired fasting glucose status (IFG), reflecting the worsening of the metabolic profile in the transition from NFG to IFG status. In women, the prevalence of IDF-defined MetS increased from 18% in those with NFG to 59% in those with IFG and 69% in those with diabetes (DM), and in men, an increase from 6% in NFG to 28% in IFG and 34% in DM was seen. Across all categories of glycaemic status, the revised NCEP definition identified more individuals with MetS than the IDF definition. Statistically, significant differences (*P* < 0.05) were observed between NCEP and IDF-defined prevalences of MetS among the males in the IFG and DM categories, but among women, the differences observed were statistically not significant (*P* > 0.05). 

The prevalence of components of MetS in the cohort is given in Table 2. Hyperglycemia (65.1%) and hypertension (57.8%) were the two most frequent MetS components in the total cohort, and no significant gender difference was observed in them. Hypertriglyceridemia was commoner in men than in women (33% versus 27.6%; *P* < 0.05). Remarkable gender difference was observed in the prevalence of abdominal obesity (women 70.2%, men 35.1%, *P* < 0.001) and in the prevalence of low HDL-C (women 51.5%, men 7.4%, *P* < 0.001). In the cohort, 19% had at least one MetS component, 57% had three components, 35% had four, and 8% had all five.

**Figure 2:**
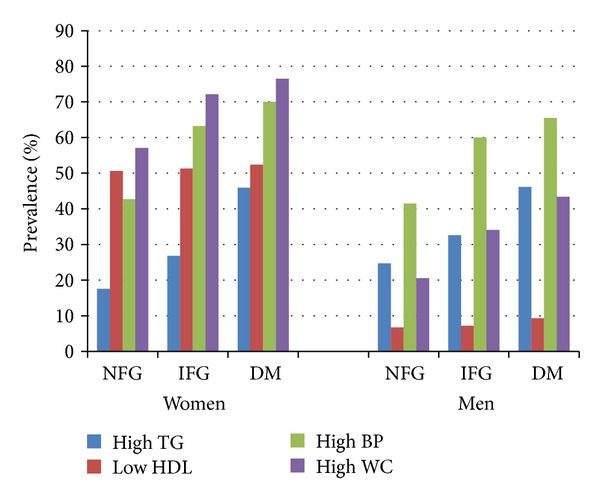
Gender-specific prevalences of components of MetS categorized by glycaemic status (NFG: normal fasting glucose, IFG: impaired fasting glucose, and DM: diabetes mellitus).

The prevalence of individual components of MetS significantly increased (*P* < 0.05) with the worsening glycaemic status (Figure 2) except low HDL-C which had the minimal change with declining glycaemic control in both genders. Significant gender differences were observed in abdominal obesity and in low HDL-C levels across all categories of glycaemic status. The highest prevalences of abdominal obesity, hypertriglyceridemia, and hypertension were seen in diabetic subjects when compared to normoglycemic or IFG subjects.

Table 3 contains a comparison of anthropometric characteristics and metabolic variables of groups with MetS by revised NCEP and IDF criteria. 21% of the men and 7% of the women missed out by the IDF definition were identified by R-NCEP definition. In both genders, individuals identified only by R-NCEP definition were less obese and had significantly lower levels of waist circumference and BMI (*P* < 0.001) than their counterparts identified by both IDF and R-NCEP definitions. However, more adverse mean levels of metabolic risk factors were observed in both men and women in the discrepant group which was identified with R-NCEP definition only, although some of these observed differences were statistically not significant. Men diagnosed with R-NCEP definition had significantly higher mean levels of serum triglycerides (*P* < 0.000), low HDL-C (*P* < 0.05), diastolic blood pressure (*P* < 0.03), and fasting blood glucose (*P* < 0.02), while the differences observed among women in the discrepant group were statistically not significant (*P* > 0.05). However, in both men and women in the discrepant group, insulin resistance defined by HOMA-IR was significantly lower (in men *P* < 0.001; in women *P* < 0.01) than in subjects identified by both IDF and revised NCEP definitions.

## 4. Discussion

South Asians are a high-risk population with respect to diabetes and CVD, and the numbers are constantly rising [20]. The prevalence of MetS in this urban Sri Lankan population is high regardless of any definition used (revised NCEP 46% and IDF 39%). In a recent study, a MetS prevalence of 24.3% by IDF definition has been reported for a nationally representative sample of Sri Lankans which included both urban and rural populations [21]. Our results are comparable with the data reported for urban populations of other South Asian countries. Using different definitions, MetS prevalences ranging from 18% to 46% have been reported from urban Karachi, Pakistan [22], from 18% to 41% prevalence from urban India [23, 24] and from 20% to 23% prevalence from eastern Nepal [25]. Rapid industrialization and urbanization and the consequent changes in the form of sedentary lifestyles could partly explain the high prevalences in urban populations.

Our results indicated marked heterogeneity in the prevalence of MetS according to gender. This is especially evident in the IDF-defined prevalence, which among men is exactly half that among women (23% versus 46%, *P* < 0.001). This gender disparity might be partly explained by the significantly higher prevalence of abdominal obesity in women (70% versus 35%, *P* < 0.001). Abdominal obesity is strongly recognized as the most important correlate of insulin resistance and MetS [26] and has a strong association with dyslipidemia, hyperinsulinemia, hypertension, and impaired fibrinolytic capacity [27, 28]. Although the prevalence of abdominal obesity in men is half of that in women, prevalences of other components of MetS were remarkably comparable between genders with the exception of low HDL-C. In women and men, respectively, hyperglycemia (64% versus 67%; *P* > 0.05), hypertension (59% versus 56%; *P* > 0.05), and dyslipidemia (raised triglycerides 28% versus 33%; *P* < 0.05 and low HDL-C 52% versus 7%; *P* < 0.001). These findings may indicate that especially among men, the currently accepted Asian-specific cut-off of 90 cm for waist circumference is not adequate to describe the effects of abdominal obesity. Although a markedly lower prevalence of low HDL-C is seen among men in our study, their mean HDL level was 48.9 mg/dL ± 4.9, only slightly below the cut-off of 50 mg/dL. However, this low prevalence is likely to be partly related to alcohol consumption and work-related physical activity. 61% of the males consumed alcohol as opposed to 4.5% of the females. Moderate-to-high intensity physical activity was also commoner among the males (84% versus 78%). 

Both IDF and revised NCEP-defined prevalences of MetS increased significantly with worsening glycaemic status, especially with the progression from normal to impaired fasting glucose status. Similar results have been documented in NHANES III study [6], in which the revised NCEP-defined prevalence of MetS increased from 25.8% to 71.3% in the progression from normal to impaired fasting glucose status. IFG identifies a high-risk group of people who are more insulin resistant and have an elevated risk for progression into type 2 diabetes and CVD. In our study, IFG was more prevalent among men than among women (45% versus 40%), which is in agreement with previous studies which demonstrated a role of sex hormones on blood glucose metabolism [29]. Although altered glucose metabolism is associated with anthropometric variables such as overweight and abdominal obesity [30], the prevalence of abdominal obesity in men with IFG was 34% as opposed to 72% in women with IFG. In the DM group, a similar trend was observed with a prevalence of 43% and 76% in men and women, respectively. The relationship between anthropometric variables, abdominal obesity in particular, and dysglycemia is also known to be modulated by gender with the female gender demonstrating a higher association than males [31]. This could partly explain the lower prevalence of abdominal obesity in males in our study despite the higher prevalence of dysglycemia. 

In the total cohort, the IDF definition did not identify 21% of the men and 7% of the women identified with the revised NCEP definition of metabolic syndrome. One distinctive feature in our study was that this discrepant group had a more adverse metabolic profile, despite having a lower BMI and waist circumference values than those identified with MetS by both the IDF and revised NCEP definitions. Accordingly, IDF definition failed to identify patients at high risk of type 2 diabetes or CVD, especially among males in whom the adverse metabolic profile was statistically significant. Similar to our findings, in a study among the Koreans, the IDF definition failed to identify 44.9% of men and 16.6% of women as having MetS according to the revised NCEP definition [32]. Those missed out by the IDF criteria were predominantly males who had a lower BMI and waist circumference but had higher cardiometabolic risk than those diagnosed with both criteria. Their blood pressure, glucose, total cholesterol, and triglycerides levels were more adverse. Similar results were found in other studies among Asians [33, 34], although in certain other cohorts IDF definition has been better in identifying individuals with higher cardiometabolic risk [35, 36]. 

This discrepancy was caused mainly by the waist circumference criterion which is a prerequisite for the diagnosis of MetS based on IDF definition. In contrast, the revised NCEP criteria consider abdominal obesity as one of the equally weighted criteria. Abdominal obesity has a strong association with insulin resistance [37], and waist circumference is strongly correlated with HOMA-IR [38, 39]. Accordingly, in our study, the discrepant group which had waist circumferences below the specified cut-off levels had lower insulin resistance as indicated by HOMA-IR than their counterparts identified by both IDF and revised NCEP definitions. Despite the wide use of HOMA-IR, no consensus has been reached regarding the HOMA-IR cut-off value for identifying subjects with IR [40]. Radikova et al. [40] selected the 75th percentile which corresponded to HOMA-IR values of 3.04 and 2.29 for diabetic and nondiabetic populations, respectively, to define insulin resistance. In other studies, HOMA-IR > 3.8 corresponding to the 90th percentile of distribution in healthy adult Spanish population [41], HOMA-IR > 1.78 corresponding to the lower limit of the top quintile of distribution for an Iranian population [42], and HOMA-IR > 1.7 in Japanese adults have been used to define insulin resistance [43]. Racial and ethnic variabilities in the HOMA-IR cut points to diagnose IR are probable [44]. The discrepant group in our study included a metabolically obese, normal weight (MONW) group of individuals who are predisposed to diabetes and CVD like people with overt obesity. According to Ruderman et al. [45] MONW individuals are very common in the general population and they probably represent one end of the spectrum with metabolic syndrome. Therefore, the revised NCEP definition seems to be more appropriate for the diagnosis of MetS in this cohort of Sri Lankans given that the IDF definition did not identify high-risk individuals who lack abdominal obesity; nevertheless showed a clustering of metabolic risk factors. 

## 5. Conclusions 

In conclusion, regardless of the MetS definition used, prevalence of MetS is high in this urban cohort of Sri Lankans. Prevalence of MetS and its components increased significantly with declining glycaemic control. The marked gender difference in prevalence estimates was attributed mainly to abdominal obesity. Our findings stressed the need for determining the suitability of the currently accepted Asian-specific cut-offs for waist circumference in Sri Lankan adults. Early identification of metabolic abnormalities and appropriate intervention may be of primary importance in this population. Our study further contributes to the mapping of prevalences of MetS among South Asians who are high-risk populations with respect to diabetes and CVD.

## Figures and Tables

**Table 1:** Prevalence of metabolic syndrome by IDF and revised NCEP ATP III definitions by gender and age groups.

	*n*	IDF	Revised NCEP ATP III
		% (95% CI)	% (95% CI)
Total	2985	38.9 (37.18–40.67)	46.1 (45.8–46.4)
Women	1627	45.8 (43.38–48.22)	56.1 (55.8–56.5)
Men	1358	22.9 (20.72–25.19)	33.9 (33.6–34.2)
Age (years)			
Women			
35–44 yrs	274	37.0 (31.30–42.70)	44.2 (38.3–50.0)
45–54 yrs	630	50.9 (47.14–54.88)	58.6 (54.8–62.4)
55–65 yrs	723	55.5 (52.38–65.45)	65.7 (62.2–69.1)
Men			
35–44 yrs	241	17.8 (13.39–23.06)	29.8 (24.0–35.6)
45–54 yrs	509	26.5 (23.20–30.96)	34.9 (30.8–39.1)
55–65 yrs	608	27.9 (24.50–31.63)	36.9 (33.0–40.7)

CI: Confidence interval.

Prevalences have been adjusted for the Ragama population using equal weights for each group in each gender.

**Table 2:** Prevalence of components of metabolic syndrome.

	*n*	Waist circumference (men ≥ 90 cm women ≥ 80 cm) % (95% CI)	Serum TG (≥150 mg/dL) % (95% CI)	Low HDL-C (men ≤ 40 mg/dL women ≤ 50 mg/dL) % (95% CI)	Hypertension (≥130/85 Hg mm) % (95% CI)	Fasting blood glucose (≥100 mg/dL) % (95% CI)
Total	2985	54.4	31.0	31.6	57.8	65.1
		(52.61–56.18)	(29.34–32.65)	(29.93–33.26)	(56.02–59.57)	(63.39–66.81)
Women	1627	70.2	27.6	51.5	58.9	63.9
		(67.97–72.42)	(25.42–29.77)	(49.07–53.92)	(56.5–61.29)	(61.56–66.23)
Men	1358	35.1	33.0	7.4	56.3	66.5
		(32.56–37.63)	(3.49–35.50)	(6.00–8.79)	(53.66–58.93)	(63.98–69.01)

CI: confidence interval and HDL-C: low density lipoprotein cholesterol.

Prevalences have been adjusted for the Ragama population using equal weights for each group in each gender.

**Table 3:** Anthropometric characteristics and metabolic variables of groups with metabolic syndrome by IDF and revised NCEP ATP III definition.

	IDF and R-NCEP	R-NCEP only	
	(men 79%, women 93%)	(men 21%, women 7%)	*P*
	mean ± S.D.	mean ± S.D.	
Age (years)			
Men	53.7 ± 7.6	52.7 ± 5.3	>0.05
Women	53.5 ± 7.2	53.8 ± 6.9	>0.05
BMI			
Men	27.0 ± 2.9	22.4 ± 1.7	<0.001
Women	26.9 ± 3.7	22.4 ± 2.9	<0.001
Waist (cm)			
Men	98.0 ± 6.5	84.4 ± 4.4	<0.001
Women	91.5 ± 7.7	75.2 ± 3.7	<0.001
Systolic blood pressure (Hg mm)			
Men	148.7 ± 21.5	151.9 ± 19.2	>0.05
Women	143.6 ± 22.8	149.6 ± 23.8	>0.05
Diastolic blood pressure (Hg mm)			
Men	89.2 ± 11.7	91.9 ± 8.7	<0.03
Women	84.4 ± 12.1	87.4 ± 11.4	>0.05
Fasting blood glucose (mg/dL)			
Men	130.9 ± 44.7	148.2 ± 61.6	<0.02
Women	129.4 ± 48.7	133.0 ± 50.7	>0.05
Total cholesterol (mg/dL)			
Men	208.3 ± 38.2	219.8 ± 45.1	>0.05
Women	217.5 ± 43.8	228.5 ± 51.2	>0.05
Triglyceride (mg/dL)			
Men	183.4 ± 79.1	222.0 ± 87.7	<0.000
Women	151.6 ± 73.4	155.5 ± 60.0	>0.05
HDL cholesterol (mg/dL)			
Men	48.1 ± 5.5	46.5 ± 6.3	<0.05
Women	49.2 ± 4.1	48.8 ± 4.4	>0.05
HOMA-IR			
Men	4.05 ± 5.7	2.49 ± 1.5	<0.001
Women	3.74 ± 3.2	2.89 ± 1.7	<0.01

HOMA-IR: homeostasis model assessment for insulin resistance.
